# Fishing Long-Fingered Bats (*Myotis capaccinii*) Prey Regularly upon Exotic Fish

**DOI:** 10.1371/journal.pone.0080163

**Published:** 2013-11-27

**Authors:** Ostaizka Aizpurua, Inazio Garin, Antton Alberdi, Egoitz Salsamendi, Hans Baagøe, Joxerra Aihartza

**Affiliations:** 1 Department of Zoology and Animal Cell Biology. Faculty of Science and Technology, University of The Basque Country, UPV/EHU, Leioa, The Basque Country; 2 The Natural History Museum of Denmark, Zoological Museum, Copenhagen, Denmark; University of Western Ontario, Canada

## Abstract

The long-fingered bat *Myotis capaccinii* is a European trawling bat reported to feed on fish in several Mediterranean locations, but the ecological circumstances of this behavior have not yet been studied. To elucidate the importance of fishing in this bat's diet, we evaluated the frequency and seasonal variation of fish remains in 3,000 fecal pellets collected from *M. capaccinii* at a nursery roost in Dénia (Eastern Iberian Peninsula) in 2008, 2009, and 2010. Fish consumption occurred evenly throughout the year. All otoliths found in feces were identified as belonging to the surface-feeding fish *Gambusia holbrooki*. Measuring otoliths, we estimated that the mean size of consumed fish was significantly smaller than the mean measured for available fish, suggesting that the long-fingered bat's relatively small body may constrain its handling of larger prey. Of note, one bat had eaten 15 fish, showing that fish may be a locally or seasonally important trophic resource for this species. By capturing 15 bats and radio-tracking the four with the most fish remains in their droppings, we also identified fishing areas, including a single fishing ground comprising several ponds within a golf course. Ponds hold a high density of *G. holbrooki*, suggesting that the amount of fish at the water surface may be the principal factor triggering fishing. The observed six-fold increase in percentage of consumed fish across the study period may be related to recent pond-building in the area. We discuss whether this quick behavioral response is a novel feature of *M. capaccinii* or an intrinsic feature that has erupted and faded locally along the species' history.

## Introduction

Bats exhibit an unparalleled trophic diversity among living mammalian orders, as their diet includes insects, fruit, leaves, flowers, nectar, pollen, blood, and other vertebrates [1], [2]. Insectivory has been widely accepted as the original chiropteran feeding behavior [3], with foraging on food other than insects seemingly evolving from insectivorous ancestors. Piscivory, a form of carnivory specialized to consuming fish, likely evolved from “trawling”, a specialized form of insectivory in which bats fly low above the water and gaff insects with their hind feet [4]–[6]. Piscivory is the primary feeding strategy for few bat species; only *Noctilio leporinus* and *Myotis vivesi* can be defined as truly piscivorous [7]–[9]. Still, though predominantly insectivorous, some other bats also prey on fish to varying degrees, e.g. *Myotis macropus, M. albescens, M. macrotarsus, M. ricketti, M. stalkeri, M. capaccinii, Megaderma lyra, Noctilio albiventris*, and *Nycteris grandis* (e.g. [4], [10]–[16]).

The long-fingered bat (*M. capaccinii*) is the only one of the three European trawling bat species known to catch fish [17]. It is restricted to the Mediterranean and the Middle East [18] and hunts over water bodies [19], [20]. In those habitats, it mainly preys upon arthropods [21]–[23], but fishing has also been reported as an uncommon and temporally irregular behavior in the Western Mediterranean [4], [21], [23]; in contrast, a high frequency of fish remains was reported in fecal pellets from *M. capaccinii* in the Levant in winter [22]. Overall, the long-fingered bat is depicted as a predominantly insectivorous species that is able to fish under as-yet undetermined ecological circumstances.

Two non-exclusive hypotheses have been proposed to explain piscivory by *M. capaccinii* in the Mediterranean. Levin *et al.* [22] linked this behavior to recent environmental changes produced by human activities: namely, the introduction of exotic fish species which may become very abundant in the bat's foraging habitats. Previous studies pointed out that fish consumed by other bats—e.g. *Noctilio leporinus*—were also exotic species [24]. On the other hand, Aihartza *et al.* [17] proposed that the occasional fishing observed in the Western Mediterranean might be tied to seasonal factors affecting changes in prey availability, and suggested that the dry season would create shallower waters with very high fish densities, which in turn could trigger fishing activity of long-fingered bats. In that sense, dry summer months in the Iberian Peninsula's Western Mediterranean coast would offer a suitable trigger scenario. Accordingly, several other fishing bats show seasonal variation in degree of fish consumption, in most cases concentrated in the dry season [16], [24], [25].

Laboratory observations have shown that bats are not able to detect prey under water but are very sensitive to surface disturbances [1]. Therefore, long-fingered bats are expected to feed on fish that swim near the water surface and occasionally break the surface layer. In the Mediterranean, cyprinodontiform fish are surface feeders that often break the surface to hunt mosquitoes. Consistently, fish remains found in feces of *M. capaccinii* were assigned to cyprinodontiform species in both the Iberian Peninsula (unidentified species, Aihartza *et al.* [4]) and the Levant (*Gambusia affinis*, Levin *et al.* [22], but see Biscardi *et al.* [23]).

Furthermore, some prey might be outside the size range that bats can handle, mainly as a consequence of their size and hardness [26]–[28]. Although insectivorous bats tend to catch the largest available insects [16], [29], [30], and the long-fingered bat is no exception [21], the weight of vertebrate prey may entail an additional constraint. Within sizeable limits, physical and energetic consequences of fish weight are commonly negligible for large predators such as the osprey *Pandion haliaetus* [31], but they do represent a constraint for the smaller kingfishers: the belted kingfisher *Megaceryle alcyon alcyon* preys on fish less than 140 g [32], whereas the common kingfisher *Alcedo atthis* has an upper limit around 35 g [33]. For *M. capaccinii* (6–10 g) preying on fish 1–3 cm long [23], wing-loading would increase 13.6% on average for each gram of prey, and carrying a 3-g fish would force the bat to double its flight speed to stay airborne (calculated after Norberg and Rayner [5]). The ground effect would facilitate transport of prey items near the water surface, but not farther up; thus, due to the aerodynamic handicap, *M. capaccinii* would likely discard the largest prey.

The main goal of this study is to characterize predation on fish by the primarily insectivorous bat *M. capaccinii*, focusing on phenology of such behavior and consumed prey. Specifically, we aim to test whether piscivory occurs seasonally, mainly associated with the dry season, fitting the "fish-abundant and shallow pond scenario"; to identify prey species and size, in order to elucidate any size-driven selection; and to locate the bat's fishing grounds, in order to investigate the appearance and conditions of its fishing behavior in the wild.

## Materials and Methods

### Study area

The study was carried out in Dénia (Eastern Iberian Peninsula), 38.82° N 0.06° E, a region characterized by a Western Mediterranean climate [34] with an extreme drought in summer. Its riparian zones and water bodies have been profoundly modified in recent decades for human activities, with many river canalizations and the spread of irrigation canals. High agricultural pressure has reduced natural river flows and groundwater levels, negatively impacting the area's aquatic ecosystems. Our studied roost in the Punta de Benimáquia limestone cave (Montgó Natural Park) is used by one of the three colonies for which fish-eating behavior in *M. capaccinii* was previously described [4], [22], [23].

### Ethics statement and conservation constraints

*Myotis capaccinii* is a threatened species. Its overall status is “vulnerable” according to IUCN criteria for risk of extinction [35], and it is classified as “endangered” in the Spanish Catalogue of Threatened Species. The average number of long-fingered bats in the Punta de Benimáquia colony was 64 individuals (unpublished data). Our study was designed to minimize potentially damaging disturbances to the small population, and hence obtained the phenology of fishing from passively collected feces.

Animal capture and handling protocols followed established guidelines for treatment of animals in research and teaching [36], met Spanish legal requirements, and were approved by the Regional Government of Valencia (2010/20964) and *a posteriori* by the Ethics Committee at The University of the Basque Country (Refs. CEBA/220/2012/AIHARTZA and CEBA/221/2012/AIHARTZA).

Feces were collected from a passive collector set at the roost, and samples were always taken at night after bats had emerged, with a collection frequency at the minimum required for this type of study. In the June 2010 capture, bats were released into the roost after body measurement and feces collection. To minimize stress, retention time never exceeded 90 minutes. Before their release, we checked the bats' ability to move properly and whether the transmitter interfered with flight [37]. The transmitter eventually fell off after 11–23 days (J. Aihartza, pers. obs.). One year after radio-tagging, bats do not appear to suffer major long-term effects of carrying transmitters within the 5% body mass rule [38]. In addition, extensive radio-tracking studies have been carried out on this species [19], where animals were followed for a long time with no signs of stress or affection.

### Phenology of fishing

Feces of *M. capaccinii* were collected during the time the bats occupied the cave in 2008, 2009, and 2010: once every fortnight in 2008 (10 samples), and weekly in 2009 (32 samples) and 2010 (20 samples). Feces were passively gathered below the main colony group in a collecting net (approximately 1 m^2^), which was replaced and relocated after each sampling. We analyzed 50 pellets from *M. capaccinii* per sampling date except in six cases for which fewer than 50 pellets were found. In April 2010, droppings were stored jointly in alcohol and the contents of individual pellets intermingled; thus, only the overall presence of fish remains could be ascertained for that month.

Feces were soaked in water prior to analysis and teased apart using two dissecting needles under a magnifying lens. Arthropod remains were identified with the aid of Barrientos [39], McAney *et al.* [40], and a reference collection. Only feces with remains of chironomid pupae were attributed to *M. capaccinii*, as they are heavily consumed by this bat [21]–[23], and none of the other species roosting in the same cave (*Myotis myotis*, *M. blythii*, *M. emarginatus*, *M. escalerae*, *Miniopterus schreibersii*, and *Rhinolophus ferrumequinum*) are known to feed on them [41]–[45]. Nevertheless, the presence of fish remains was also checked in pellets without chironomid pupae, with negative results in all cases. Fishing activity in each sampling period was assessed as percentage occurrence of fish remains in feces, i.e. scales, otoliths, and other bones identifiable as belonging to fish [4], [22], [23]. Month and season averages were calculated by the average percentage of the presence of fish in each sampling period. Consumption differences between months and between years were tested using Kruskal-Wallis H tests (K-W). To test whether fishing incidence was higher during the dry season, we compared the relative importance of fishing between the dry and the wet season using a Mann-Whitney U test (M-W). Seasons were defined based on monthly precipitation data from Alacant (the closest climatological station) obtained through the Spanish Meteorological Agency (AEMET; available at www.aemet.es). Using a monthly precipitation threshold of 30 mm, June, July, and August were included in the dry season, and the remaining months in the wet season. Differences in otolith length were tested using Student t-tests (*t*-test).

### Prey identification and size assessment

Dietary studies of piscivorous bats, including *M. capaccinii*, have traditionally identified prey species and estimated their size based on size and shape of fish scales [4], [13], [22]–[24], [46]–[48]. However, species-level identification is not always possible with this method, and the reliability of body size estimates is at least questionable if the scales do not correspond to a specific body part (e.g. lateral line) [49]. Alternatively, seabirds' prey species have been identified using otoliths (e.g. [50]–[56]), the thickest structures in the body of teleost fishes [57]. *In vitro* [58] and *in vivo* [59] experiments have shown that they are hardly digested, and hence are often the only remnant of bony fishes found in predators' feces. Moreover, fish otolith morphology is species-specific, so it offers a reliable tool for prey identification to species level [60] as well as size estimation (e.g. [52], [55], [56], [61]–[65]). Significant aspects of this bat's fishing ecology, such as the minimal prey items consumed and the biomass of prey items, can be readily inferred by visual inspection of otoliths.

Fish scales in feces were identified to order level following Elvira [66] and by comparing to a reference collection, whereas we relied on otoliths for species-level identification. Eleven otoliths found in feces were discarded due to excessive digestive erosion or to appearance of two types of otoliths (sagitta and lapillus) in the same sample. As a reference collection, we used otoliths gathered from the cyprinodontiform fish Spanish toothcarp *Aphanius iberus* (endemic), Valencia toothcarp *Valencia hispanica* (endemic), and eastern mosquitofish *Gambusia holbrooki* (exotic). Otoliths of the former two species were obtained from dead and polygenic animals not suitable for reintroduction, provided by the Regional Ministry of the Environment, whereas otoliths of *G. holbrooki* were obtained from free-living animals as follows.

We captured 100 eastern mosquitofish in water bodies of the study area. Their body length was measured to assess the size-range of the species in the region and to build the correlation between otolith and fish body length. We extracted the sagittae and lapilli from 50 variously sized individuals previously sacrificed by cervical dislocation [67] and measured their greatest length from anterior tip to posterior edge [68] using a stereomicroscope (Nikon SMZ 1500) equipped with a digital camera (Nikon DS, 5 Mpx).

As no difference between right and left otoliths was observed (*t*-test: t_1,49_ = 0.650, p>0.050), a single otolith (right or left) from each specimen was randomly used to build the exponential regression model between otolith size and body length [62], [65], [68]. The length-body mass relationship of fish was calculated using body length and mass measurements of 100 fresh eastern mosquitofish, which were fitted to the power function W = *a* * L*^b^*, where *W* is the fish body mass, *a* the intercept of the regression line, *L* the fish length, and *b* the regression coefficient [69].

Otoliths found in feces were measured by the same procedure as those extracted from fresh fish. Biomass of consumed prey was estimated using the length-body mass power function built as described above. Size and body mass of available fish were compared with the values of consumed fish using the M-W test. All statistical analyses were carried out using SPSS 20.0.0 statistical software (SPSS Inc., Chicago, IL, USA). The format for reporting mean values and statistical test results was: mean values (mean ± standard deviation, n  =  sample) and statistical tests (test type: statistic_numerator d.f., denominator d.f_, p-value)

### Localization of fishing grounds

To investigate whether *M. capaccinii*'s fishing activity was triggered only by the specific condition of shallow water with high fish density, in June 2010 we used a harp trap (modified from Tuttle [70]) to capture 15 long-fingered bats as they entered the cave after their first foraging bout, approximately 2 hours after emergence. The bats were sexed, weighed, and aged before being kept individually in cloth bags until they defecated. The content of collected pellets was inspected in the field under a dissecting microscope. The four bats whose feces contained the most fish remains were tagged with radio-transmitters (0.45 g; Pip II, Biotrack Ltd., Dorset, UK) using surgical cement (Skinbond, Smith and Nephew, Largo, Florida, USA). The transmitter's mass never exceeded 5% of the bat's body mass, as recommended by Aldridge and Brigham [71], so fishing behavior was presumably unaltered by the load of the transmitter. Bats were tracked by car and on foot using triangulation (for initial broad-scale localization) and homing-in (for subsequent fine-scale localization) methods, with the aid of radio receivers (1000-XRS, Wildlife Materials Inc., Carbondale, USA, or FT-290RII, Andreas Wagener Telemetrieanlagen, Köln, Germany) and Yagi antennae.

Fish species and abundance were analyzed in putative fishing areas determined by the tracked bats' activity. Presence and activity of long-fingered bats, both tagged and untagged, was observed using an HD video camera with infrared imaging capability (HDR550, Sony Corporation, Tokyo, Japan) and an ultrasound detector (D1000X, Pettersson Elektronik AB, Uppsala, Sweden). The species was identified by its characteristic flight pattern and echolocation signals, unique among the bat species in the area. Fishing attempts were confirmed using a low-light high-speed video camera (HiSpec, Fastec Imaging Corporation, San Diego, California, USA), with recordings aided by infrared light torches (IREL-45).

## Results

### Phenology of fishing

**Figure 1:**
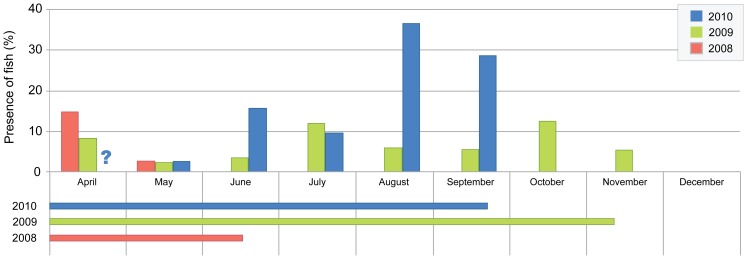
Percentage of fish remains in *Myotis capaccinii* feces. Horizontal bars show the time period that bats were in the cave. Note: question mark in April indicates that although fish remains were detected, frequency percentage could not be calculated due to sample degradation.

The amount of feces analyzed each year depended on the time bats spent in the cave and the frequency of dropping collection. Thus, we analyzed 409 pellets in 2008 (March–June), 1,600 in 2009 (April–November), and 1,050 in 2010 (March–September). Traces of fish were observed in feces every year (Figure 1), with no significant differences in amount between months (K-W: H_7,61_ = 9.248, p = 0.235). In 2008, fish was consumed in two of the three months that bats remained in the cave; in 2009, fish remains were found in all periods, showing the highest peaks in July and October; and in 2010, fish was consumed every month, with the highest peaks in August and September. There was no difference in the relative importance of fish in the diet of long-fingered bats between the dry (11.15±14.6%, n = 32) and the wet (10.39±15.0%, n = 30) season (M-W: U_1,61_ = 492, p = 0.864). The presence of fish remains in feces did differ across the years (K-W: H_2,68_ = 9.123, p = 0.010), with percentage of occurrence rising from 3.1% in 2008 to 6.3% in 2009 and 18.0% in 2010.

### Prey identification and size assessment

**Figure 2:**
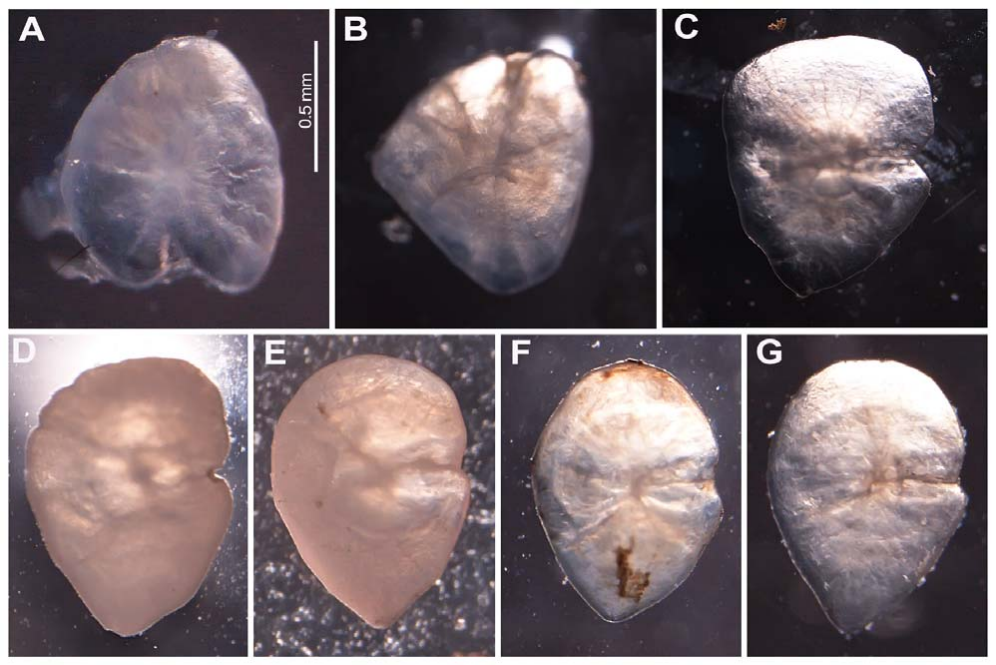
Sagittae otoliths of cyprinodontiform species in Dénia. (A) *Aphanius iberus*, (B) *Valencia hispanica*, (C) *Gambusia holbrooki*. (D–E) Sample of otoliths in *Myotis capaccinii* feces. All images are at the same scale.

The quantity of fish remains per pellet varied from a single scale to 100% of the pellet. All fish scales found in feces were assigned to the order Cyprinodontiformes. We recovered 97 otoliths from feces and used the best-preserved (73 sagittae and 13 lapilli) for prey identification and size assessment; all belonged to *Gambusia holbrooki* (Figure 2).

**Figure 3:**
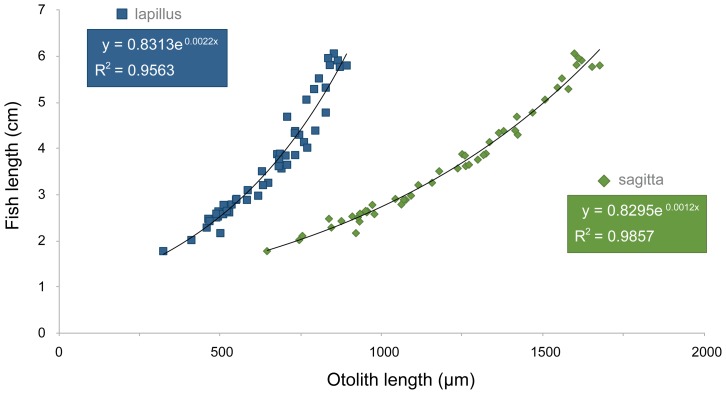
Relationship between length of otoliths (µm) and body length (cm) of the eastern mosquitofish *Gambusia holbrooki*.

**Figure 4:**
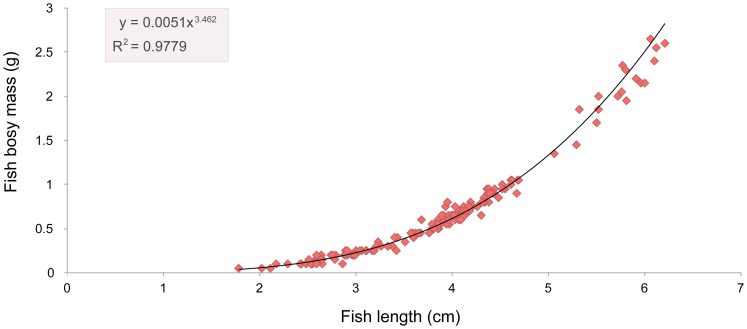
Relationship between body length (cm) and body mass (g) of the eastern mosquitofish *Gambusia holbrooki*.

The relationship between otolith length and body length is exponential (Figure 3), as is that between body length and body mass (Figure 4). Available fish were significantly longer (M-W: U_1,162_ = 6132, p<0.001) and heavier (M-W: U_1,162_ = 6076, p<0.001) than consumed fish (Table 1). We observed no significant difference (*t*-test: t_1,61_ = 1.29, p>0.001) between otolith sizes collected in 2009 (2.54±0.37 mm, *n* = 31) and in 2010 (2.45±0.29 mm, *n* = 31); otoliths from 2008 were not included in the analysis due to the small sample size.

**Table 1:** Summary statistics of body length and mass of available and consumed fish.

		Length (cm)			Body mass (g)		
Sample	n	Mean	SD	Range	Mean	SD	Range
Available	86	3.87	0.96	2.17 – 6.21	0.70	0.61	0.01 – 2.65
Consumed (Overall)	86	2.48	0.33	1.92 – 3.55	0.13	0.06	0.05 – 0.41
Consumed (June 2010)	51	1.74	0.11	1.42 – 2.05	0.04	0.01	0.02 – 0.06

**Consumed (Overall)** data was obtained from pellets gathered with the collector placed under the colony. **Consumed (June 2010)** data was obtained from the pellets produced by the six captured bats.

Overall, the meager amount of otoliths recovered each month did not allow us to analyze yearlong variation in size of consumed fish. However, we analyzed 56 otoliths obtained from feces of five bats captured at the roost entrance in June 2010. The mean number of otoliths per bat was 10.8±11.9, but 29 were found in droppings of one individual that had consumed at least 15 fish in a foraging bout (assuming retention of otoliths in the gut does not exceed 24 h). The mean size of those 15 fish (Table 1) was smaller than that of all consumed fish during the year (*t*-test: t_1,111_ = 17.284, p<0.001). Moreover, the cumulative mass of those 15 fish would reach 510 mg, surpassing the prey size estimated (3.55 cm and 410 mg) from the largest single otolith found in feces in 2008–2010.

### Fishing grounds

Six of the 15 captured bats showed fish remains in their fecal pellets, and the four whose feces contained the most fish remains—three lactating females and one adult male—were radio-tagged. Females were tracked for 1 week; the male specimen lost the transmitter in the roost and could not be tracked. As soon as the first tracking night, we found the bats foraging in two different rivers (Xaló and Girona) and some artificial ponds in a golf course at a 1.5-km straight-line distance from the cave. Those ponds were the only sites where syntopic foraging of the three females occurred, and were the sole hunting ground of one of them. Foraging was concentrated in two large ponds (91×32 m and 192×42 m, both approximately 3 m deep) with no surface vegetation. Furthermore, as assessed by visual inspection, fish abundance at the water surface was much higher than in the tagged bats' other foraging areas. Fish constantly broke the surface, creating a large amount of ripples. The video recordings showed high activity of long-fingered bats over the ponds, as well as bats trying to catch fish using their hind feet, some successfully.

## Discussion

We conclude that in the study area, *Myotis capaccinii* engage in piscivory evenly throughout the year, and prey upon small surface-feeding fish. Moreover, fish consumption by *M. capaccinii* in the studied colony is an extended behavior and occurs in almost all the active months of the year. The comparison of fishing incidence during dry and wet seasons showed that piscivory is not limited to the dry season, contrary to the suggestion by Aihartza *et al.* [17]; in fact, fish consumption remains almost invariable in autumn, when precipitation reaches its maximum in the Western Mediterranean region [72].

As expected, most fish remains and otoliths observed in bats' feces were unambiguously ascribed to a cyprinodontiform species, the eastern mosquitofish *Gambusia holbrooki*. Accordingly, the species consumed in the Levant was identified as *G. affinis* [22], which is similar to *G. holbrooki* in appearance and biology. In Italy, however, the fish remains were identified as the cyprinid *Alburnus alburnus*, although other species were not ruled out [23]. The eastern mosquitofish is one of the world's 100 most invasive exotic species [73] and is a serious threat to native wildlife, particularly in the Eastern Iberian Peninsula where it cohabits with two critically endangered native cyprinodontiform species, *Aphanius iberus* and *Valencia hispanica* [74], [75]. Like most cyprinodontiforms, eastern mosquitofish usually forage near the top of the water column, often taking food items from the surface using their upturned mouth [76]. Fish-eating bats can detect and identify by echolocation any potential prey exposed from the water or disturbing its surface [1], as the fish or the ripples it caused would reflect a significant echo [77].

Our size estimate for the consumed fish (1.92–3.55 cm) is similar to that reported by Biscardi *et al.* [23]. The selection of smaller fish than those generally available suggests that, even with its exceptionally large feet, the long-fingered bat's relatively small body may be energetically and/or morphologically impeded in handling larger prey. The largest fish consumed would equal 4.10% of the bat's weight, according to the relationship between fish size and body mass. In addition, a single bat consumed at least 15 small fish in a foraging bout, which could equal about 5.10% of the bat's weight. These values are far below the approximately 30% body mass increase that pregnant bats may deal with when foraging, and close to the 5% wing-load increase that laden bats are able to support without any significant decrease in maneuverability [71]. Thus, we conclude that the burden itself is not limiting. The low overall efficiency of capture observed for *M. capaccinii* both in captivity [17] and in the field (unpublished data) may reflects difficulties in seizing and handling large fish.

The frequency of fish remains among feces from the long-fingered bats trapped at the roost entrance (six of 15 individuals) gives a clue about the importance of this behavior and particularly the profitability of smaller fish. In warm environments, eastern mosquitofish may have more than one recruit per year, reaching a maximum of nine broods per female and per season [78]; thus, long-fingered bats will encounter changing prey abundances and size categories throughout the year. The significant differences in the size of consumed fish between bats captured in 2010 and overall may reflect this.

The long-fingered bat's use of ponds for foraging has been extensively recorded in the literature [19], although ponds are less preferred than rivers or canals. In general, the observed ponds met the three criteria for a preferred foraging site [20]: they were accessible (offered an open free space), prey were detectable as the water surface was smooth (lacking ripples), and hunting was profitable as fish were abundant. Furthermore, putative commuting structures such as roads and hedgerows in the vicinities of the ponds and their proximity to the roost might make the observed ponds attractive to bats. Moreover, scarcity of water bodies in the area, as well as the low quality of others, reinforces the importance of these ponds as foraging grounds for *M. capaccinii*, not only for fishing but also for hunting insects throughout the year (O. Aizpurua, pers. obs.).

Our results suggest that *M. capaccinii* may prey on fish more than was originally thought. This highly nutritional prey may be more important than we had expected for this bat, at least locally. The irregular pattern of fish consumption does not seem related to any known seasonal variation. Hence, the differential energetic profitability linked to relative abundance of different prey types might be the cause of opting for one or another. Hunting fish is energetically more expensive than hunting insects, because fish are heavier and must be dragged from the water, and capture efficiency is usually lower [8], [17]. Even so, a single fish is considerably more nutritional than a chironomid, or even a moth. Therefore, there must be a threshold of relative abundance of both prey types (e.g. very low insect availability and very high fish availability) above which fishing is more profitable than hunting insects. Accordingly, Levin *et al.* [22] proposed a low density of insects as a stimulus for fishing.

Furthermore, we cannot rule out a learning process being involved in the intensity of fishing by *M. capaccinii*. Levin *et al.* [22] suggested that such behavior might be new in this bat, initiated as a consequence of introduction of the western mosquitofish *G. affinis* in the Levant. Our findings correspond well with such a point of view, as the fishing ponds in the study area were built in 2002–2009, perhaps for the first time providing surface-feeding fish in densities high enough to be profitable. In fact, the noteworthy increase in fishing intensity from 2008 to 2010 may result from changes in prey availability and/or a learning process, as more bats within the colony might have become skilled enough to exploit a new resource. These findings apparently support the idea of Levin and colleagues [22] and may indicate that fishing is a recent behavior for *M. capaccinii* in both the Levant and the Iberian Peninsula. But does this mean that such behavior is definitely new for the species from an evolutionary point of view? Or does this merely show that the conditions making fishing feasible and/or profitable—namely, very high densities of surface-feeding fish—nowadays only occur due to anthropogenically introduced exotic fish?

Certain morphological features depict *M. capaccinii* as better adapted to fishing than the other European trawling bats *M. daubentonii* and *M. dasycneme*: namely, its large hind feet, at 10–13 mm long, are both relatively and absolutely longer than in the other two species [18], enabling it to catch larger prey; in addition, the wing membrane of the long-fingered bat starts at the tibia, which allows its feet to dip more deeply into the water. All of these adaptations and the fact that fishing has been reported in three distant places in the Mediterranean basin [4], [22], [23], suggest that fishing could have been a widespread behavior in *M. capaccinii* in other times, maybe when native surface-feeding fish—such as the currently endangered relict cyprinodontiform species within *Aphanius* and *Valencia*—and their marsh and littoral lagoon habitats were abundant in the Mediterranean. In fact, those fish might have been the primary resource from which fishing behavior could have evolved in *M. capaccinii*. We cannot discard the possibility that further research on the long-fingered bat's trophic ecology in other Mediterranean areas will reveal more cases of fishing in this species, perhaps even its predation upon other native fish resources.
